# Redundancy of terms is not an error but plays a positive role in composing search strategies

**DOI:** 10.5195/jmla.2020.780

**Published:** 2020-01-01

**Authors:** Jan W. Schoones

**Affiliations:** Collection Development Expert/Information Specialist, Walaeus Library, Leiden University Medical Center, P.O. Box 9600 C1-Q, 2300 RC Leiden, The Netherlands, j.w.schoones@lumc.nl

**To the editor,** recently, a very interesting study on errors in search strategies used in systematic reviews was published in the *Journal of the Medical Library Association* [1]. In this article, Salvador-Oliván et al. listed an impressive variation of errors in literature search strategies. Novice and expert searchers do well to keep these in mind when searching.

As to be expected, some errors have graver effects on results than others. Errors that have no effect at all on the number of results include redundant terms and repetition of morphological variants. As two reviews that I was involved in were classified under these two error types, I would like to state that these characteristics of a search strategy are not errors at all but are purposeful additions. Because a literature search is, to quote the Electric Light Orchestra, a “Livin’ Thing,” one cannot at the beginning of a search judge whether one term or the other will retrieve a crucial reference. This can only be judged after the complete search has been finalized.

To be certain that no crucial articles will be missed, redundancy of terms and repetition of morphological variants both play a role, albeit a small one. Although some redundant terms or morphological variants could be discarded in the further development of the strategy, the main value of redundancy and repetition is that at the moment in time when the search is first executed, it is uncertain whether removing a search term prior to the execution of the search will harm the results.

An example for redundancy is as follows: the first four of the following terms in a PubMed search could be easily discarded because using the fifth variation will cover all four:

“massive chronic intervillositis”[tw] OR “chronic

intervillositis”[tw] OR “chronic histiocytic

intervillositis”[tw] OR “histiocytic intervillositis”[tw] OR

“intervillositis”[tw]

But it could well be that upon judging the retrieved references, a decision would be made to do the opposite (that is, to keep the first four and delete the fifth). As it is not easy or even possible to predict whether this will happen, it is, in my view, best to keep these redundancies in, just to be sure.

So, as most search strategies will be revisited in the future, be it after a few months or sometimes years, these redundant terms and variations play a role in further developing a search and deciding whether to add or delete terms. As Salvador-Oliván et al. write, these “search errors” do not affect recall or negatively affect information retrieval with respect to either recall or precision. They do play a positive role in the composition of the search strategy.

**Jan W. Schoones,** j.w.schoones@lumc.nl, http://orcid.org/0000-0003-1120-4781, Collection Development Expert/Information Specialist, Walaeus Library, Leiden University Medical Center, P.O. Box 9600 C1-Q, 2300 RC Leiden, The Netherlands
